# A preliminary investigation of a two-step, non-invasive process to determine chronological deposition order of fingerprints and printed ink on paper

**DOI:** 10.1038/s41598-022-16740-z

**Published:** 2022-07-21

**Authors:** Roberto S. P. King, Beth McMurchie, Richard Wilson, Paul F. Kelly

**Affiliations:** 1 Foster+Freeman, Vale Park, Evesham, Worcestershire, WR11 1TD England; 2 Department of Chemistry, Loughborough University, Epinal Way, Loughborough, LE11 3TU England

**Keywords:** Techniques and instrumentation, Materials science

## Abstract

While traditional techniques have long allowed forensic investigators to positively identify fingermarks on documents of interest, understanding the chronological sequence of events that led to their deposition is still seen as a ‘holy grail’ for forensic examinations. By way of example, the question of whether a mark is above or below printed text is crucial. The work herein reveals that a novel application of a recently established fingermark development technique readily allows such differentiation. The process in question allies forensic gelatin lifters with RECOVER, a development system that hinges on the polymerisation of disulfur dinitride. While the latter was specifically developed in its current form for the retrieval of prints from metal surfaces exposed to extreme conditions or washing, its ability to target surface effects allows for visualisation of surface interactions on forensic gelatin lifts. Crucially, in doing so the order in which the lifted material was originally deposited is also revealed. This, therefore, permits clear elucidation of the order of deposition of printed text and fingermarks—and does so both rapidly and in a non-invasive way. This long sought-after capability has the potential to revolutionise forensic document examinations.

## Introduction

Although fingermarks are the oldest form of forensic identification, they are still a cornerstone of a forensic practitioners arsenal, and the concept of gelatin lifting being used to recover fingerprints has been discussed since 1913^1^. By the 1970s their design had evolved—from paper coated with a glycerol/gelatin mix, to commercially available rubber-based lifters. As a result, forensic scientists at crime scenes could readily deploy them; the appeal was, and still is, the simplicity of their use. The gelatin lifter is placed on a mark of interest and powder or dust particles adhere to the surface of the lifter. When the latter is then removed from the surface, vital information regarding this surface is easily visualised^2^. Whilst there are some issues that arise when utilising such lifters, including unwanted material being transferred, the benefits have ensured that they are still recommended for use by forensic practitioners in a range of circumstance. Such scenarios include both direct finger and footwear marks, lifting micro traces such as paint, recording patterns around bullet holes and lifting blood traces^1^. Historically, gelatin lifters have been used in order to directly lift a material of interest; however an area that has been the subject of more recent research is the idea of chemically treating these gels post lift. Examples of this have included the gelatin lifter’s ability to retrieve the minuscule metal traces from the surface of an individual’s hands that result from briefly touching the metal in question. The lift is then developed with rubeanic acid^3^. Similar results were also observed when copper was deposited via vacuum metal deposition (VMD) onto a substrate prior to being lifted and chemically treated^4^. The lifters themselves work via several different adhesion mechanisms: physical adsorption, chemical bonding, diffusion, electrostatic forces, and mechanical interlocking^2^. Whilst post processing of fingermarks lifted onto gelatin lifters has been investigated, some lifters result in heavy background staining which limits their effectiveness^5^.

Visualising latent fingermarks that have been deposited on challenging surfaces and/or in challenging circumstance is an area of much research and regular development within the forensic science community. A recent example of this progression is the aforementioned disulfur dinitride process, used primarily for developing fingermarks on metal substrates. The technique was first reported in 2008^6,7^, and whilst its development mechanisms are still not fully understood, it is only more recently through the commercialisation of the process that this method can be performed safely and in a standard forensic laboratory using the RECOVER system^8^. The RECOVER system uses the proprietary chemical, DEVELOP to produce disulfur dinitride vapours which is then selectively polymerised to (SN)_x_ on fingermark ridges^9^. This process is particularly useful for developing fingermarks which have been removed from a metal surface, through washing or heating, as the corrosion signature left on the surface of the metal, still acts as a polymerisation target for (SN)_x_ polymer^8^.

Whilst fingermark development is vital to forensic investigations, being able to add context to a suspected crime scene is an extremely important additional benefit to some development techniques. Determining the chronological sequence of fingermark residue and ink deposition onto paper has many applications^10^, particularly in fraud investigation, and it can also be pertinent information itself in a variety of cases. Much research has gone into investigating methods to determine the handling sequence and complex analytical equipment is required for any such information to be obtained. One such example is reported by Fieldhouse et al. regarding the use of electrostatic detection (ESDA)^11^. Secondary ion mass spectrometry (SIMS) has also been heavily researched^12–14^, most recently by Attard-Montalto et al. using time-of-fight secondary ion mass spectrometry (ToF–SIMS)^15^. Each of these methods requires access to and ability to operate the appropriate analytical equipment. They also require very specific conditions to be met in order to reach a possible conclusion, particularly development of the fingermarks prior to assessment, removing the possibility of non-invasive and discreet investigations. The above are all ‘manufactured’ scenarios, that whilst noteworthy are also unlikely to be encountered in a real-life setting. Therefore, it is reasonable to state that hitherto there is no technique available to forensic practitioners that would allow for the fingermark sequencing to be determined.

The work reported in this paper is designed to evaluate the use of gelatin lifters and disulfur dinitride vapours from within a RECOVER chamber in order to provide crucial evidence that could be used by forensic practitioners. As already noted, the original intended use of these vapours in RECOVER was to take advantage of the corrosion signature produced on various metal surfaces, in order to visualise fingermark deposits. What is clear with disulfur dinitride vapours is their sensitivity to surface changes. This is why the minute levels of corrosion caused by fingermark deposits on metal are so easily visualised when exposed to said vapours. With this level of sensitivity, investigation into further forensic capabilities of these vapours is logical. This study analyses the ability of gelatin lifters to retrieve information of interest from several substrates, and via exposure to disulfur dinitride vapours provide crucial additional chronological context to the fingermark deposit itself.

## Methods

All experiments were conducted with informed consent from the participants in accordance with the Declaration of Helsinki, and all donors consent to images of their developed fingermarks being used and published as part of this research. Experimental protocols were approved following Research and Development Board review at foster+freeman, UK. The datasets used and/or analysed during the current study available from the corresponding author on reasonable request and consist of images obtained from the processes discussed.

## Materials

White gelatin lifters (BVDA) were used throughout, due to ease of fingermark visualisation compared to their black or clear equivalent. These were used as per the manufacturers recommendations. A roller was used to ensure good and consistent contact between the gelatin lifter and the surface. Gelatin lifters were removed from the surface and immediately developed. A HP Colour LaserJet Pro M478f-9f was used for all printing unless stated otherwise. The RECOVER system (foster+freeman) was used to develop fingermarks on gelatin lifter surface using the S_2_N_2_ vapour method. As per the manufacturers recommendation, an R1 aliquot of DEVELOP chemical (foster+freeman) was used in each case when fuming. This quantity was selected over the alternatives (R2, R3 or R4) as it is the smallest quantity commercially available, thus limiting the chances of overdevelopment when processed in the RECOVER.

### Fingerprint sample preparation and development

Natural fingermarks were obtained from 5 donors and used throughout this research. It was ensured that the donors hands had not been washed for at least 30 min prior to fingermark deposition. Each donor completed each separate experiment by depositing a fingerprint from each finger of both hands. Fingermarks were placed in a pre-determined position on standard white copier paper, this paper was then placed into a printer and text was printed over the fingermark deposition. Further fingermarks were then placed on top of this printed text in a separate specified location. Fingermarks were either lifted immediately after deposition or left to age in situ, for up to 72 h. Gelatin lifters were then used to lift the fingermarks, which were placed either before or after printing of text had occurred. The gelatin lifter samples were then lifted carefully from the paper and placed directly in the RECOVER development chamber. Gelatin lift samples were suspended via clips from the evidence development rack within the chamber. The samples were fumed under vacuum until fingermark detail was clear, this was between 5 and 20 min, depending on speed of development, samples were removed when visible detail was present.

### Chemical enhancement of fingermarks on paper

Three common fingermark enhancement reagents were used to develop fingermarks on paper, ninhydrin, 1,2-Indandione and dizafluoro-9-one (DFO). Ninhydrin, 1,2-Indanedione and DFO solutions were comprised of petroleum ether (Fisher Scientific), ethyl acetate (Fisher Scientific), methanol (Fisher Scientific), acetic acid (Merck) along with ninhydrin (Merck), 1,2-Indandione (WA Products) and DFO (WA Products) respectively, using the formulations shown in Table 1. Zinc chloride (Merck) was also added to the 1,2-Indandione solution. The paper samples were dipped into the appropriate solution, and allowed to dry. DFO and 1,2-Indandione samples were moved to an oven at 100 °C. When developing ninhydrin samples, the oven was heated to 85 °C and 65% RH. The samples were removed from the oven after 20 min.

**Table 1:** Working solution quantities for fingermark reagents.

Solution	Respective solid (g)	Petroleum ether (ml)	Ethyl acetate (ml)	Methanol (ml)	Acetic acid (ml)
Ninhydrin	4	900	70	20	10
DFO	0.5	780	100	100	20
1,2-Indandione	0.25	1000	45	45	10

### Imaging

All images were collected using an ML Pro (foster+freeman). Gelatin samples were visualised and photographed under white light immediately. Samples were revisualised and photographed again approximately one hour after development and again 24 h after development to observe any changes. It was found that the colouring of the gel faded after 24 h which could give further clarity to the fingermark in some cases, whilst in others, detail of the fingermark faded too. Ninhydrin developed samples were visualised under white light, whilst DFO and 1,2-Indandione samples were visualised both under white light, and under blue/green light through a 550 nm filter.

## Results

**Figure 1:**
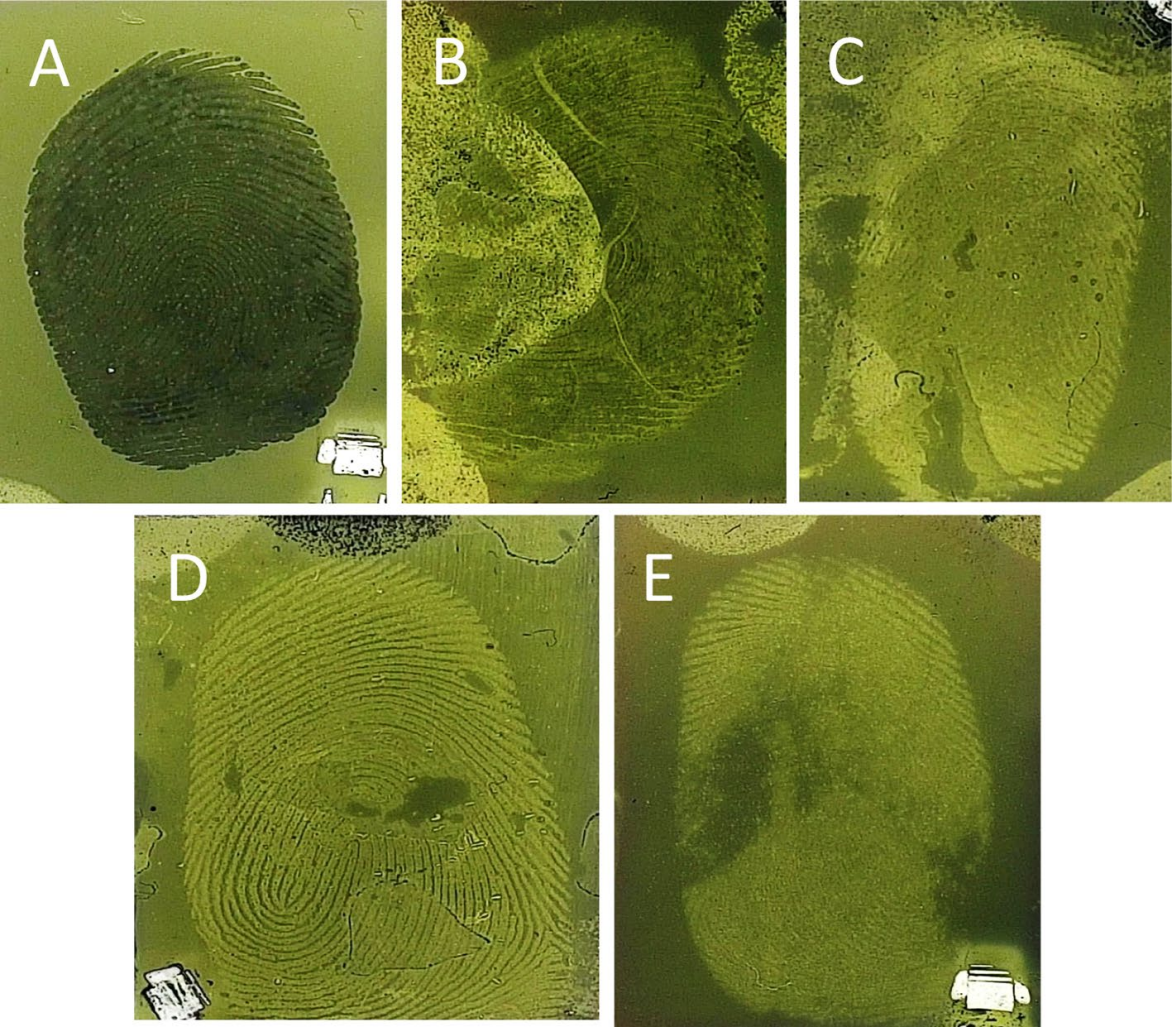
Fingermarks developed on gelatin lifters using the RECOVER system when (**A**) the finger was placed directly onto the gel surface, or fingermarks were lifted from (**B**) copper metal, (**C**) stainless steel, (**D**) glass and (**E**) paper.

Initial exploration into whether the disulfur dinitride process could develop fingermark residues on gelatin lifted fingermarks was conducted with several surface substrates. As shown in Fig. 1, a fingermark was placed directly onto a piece of gelatin lifter (A). Fingermarks were also placed on copper metal (B), stainless steel (C), glass (D) and paper (E) surfaces and these were then lifted. The fingermarks on the pieces of gelatin lifter were then developed using the S_2_N_2_ vapour process in the RECOVER chamber. Clear, detailed fingermarks can be seen on the gelatin lifter samples, corresponding to each of the target surfaces. This confirms that the polymerisation of S_2_N_2_ on the gelatin surface, to (SN)_x_, can selectively reveal fingermarks that have been lifted from various substrate types onto gelatin lifters. Whilst this in itself is an interesting result, it’s real-life application would likely be limited as each substrate discussed already has a more established ‘traditional’ method to develop fingermarks from its surface. What it did highlight however, was the potential in combining the technologies of gelatin lifting and RECOVER to provide crucial context to said established techniques, independent of the porosity of the substrate.

**Figure 2:**
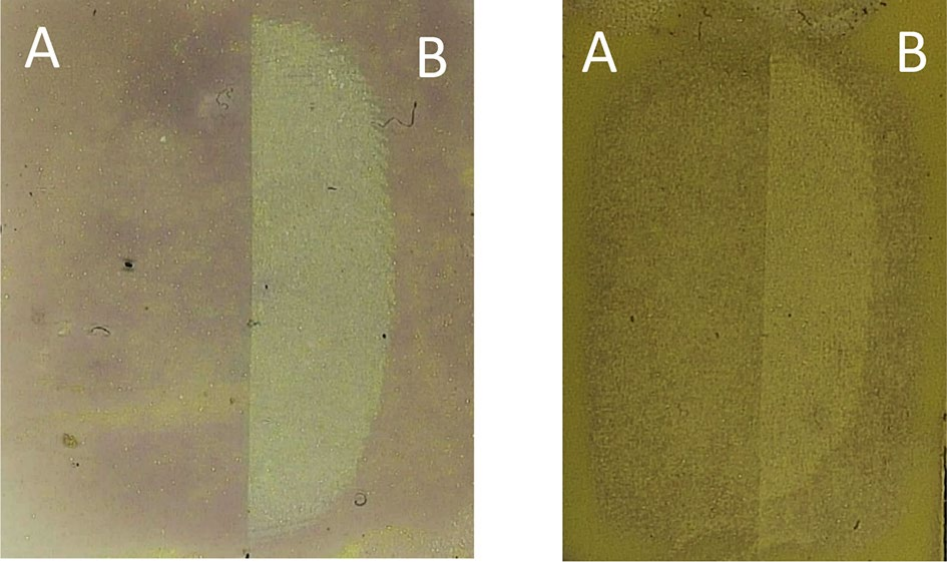
Fingermark development using RECOVER system on gelatin lifter. Fingermark deposited on paper substrate, with solid black ink printed over half of the fingermark (**A**) and the other half left uncovered (**B**). The gel on the left was lifted immediately after ink printing. The gel on the right was lifted 72 h after ink printing.

**Figure 3:**
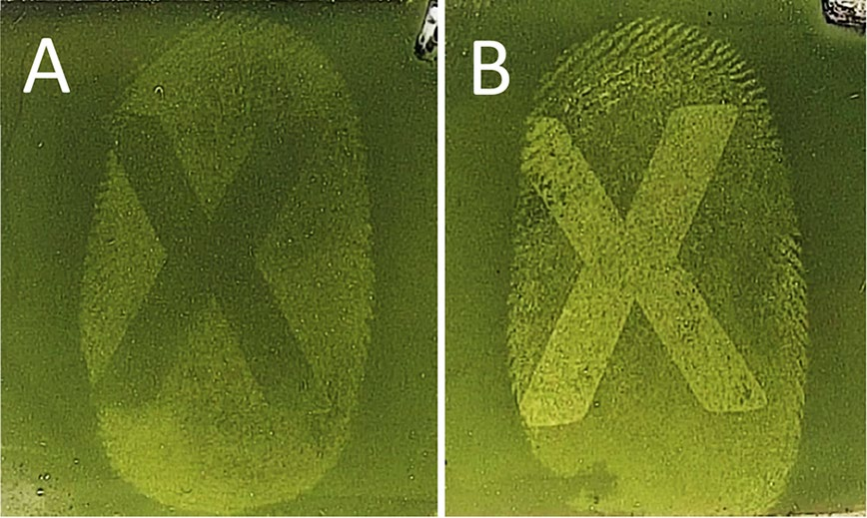
Gelatin lifted samples of fingermarks placed prior to printing an X (**A**), and on top of a printed X (**B**), developed in the RECOVER system.

Subsequently, to observe if there was any variation between fingermarks deposited prior to, or post printing, a block of black ink was printed either over an already deposited fingermark, or a fingermark was placed on top of a block of printed ink. These fingermarks were then lifted and the gelatin samples developed in the RECOVER system. Where a fingermark had been deposited on top of the block of ink, the fingermark was clearly visible when developed using RECOVER, as the gelatin lifter was able to lift the fingermark residue present on the printed paper surface. However, when developing the gelatin lifter taken from the area where a fingermark was deposited prior to printing, i.e., ink on top, the fingermark was not visible at all, as the gelatin lifter was lifting the toner surface of the paper which was concealing the fingermark that lay beneath. Figure 2 shows the result of this direct comparison of sequential variation, the right side (B) of the fingermark was not over printed with ink and development is seen, compared to the left side (A) of the fingermark which has been obscured by the ink and cannot be determined from the background of the gelatin lifter. Quality of ridge detail obtained did vary between donors, however what was more important for this study was determining the chronology of deposition, and this was possible on each occasion. The same outcome was observed when lifting the fingermarks immediately after deposition and when leaving them to age for up to 72 h, examples of these two time frames are shown in Fig. 2. Naturally blocks of ink are infrequently encountered but the results provide support for the applicability of the technique. Subsequent investigation focussed on exploring this phenomenon by printing individual letters, then progressing to blocks of text. Figure 3 shows where a bold ‘X’ was printed onto paper, half of which were printed over where a fingermark had already been placed (A), and in other half, fingermarks were placed over the already printed ‘X’ (B). In both cases, this was then gelatin lifted and the sample developed using the RECOVER system.

**Figure 4:**
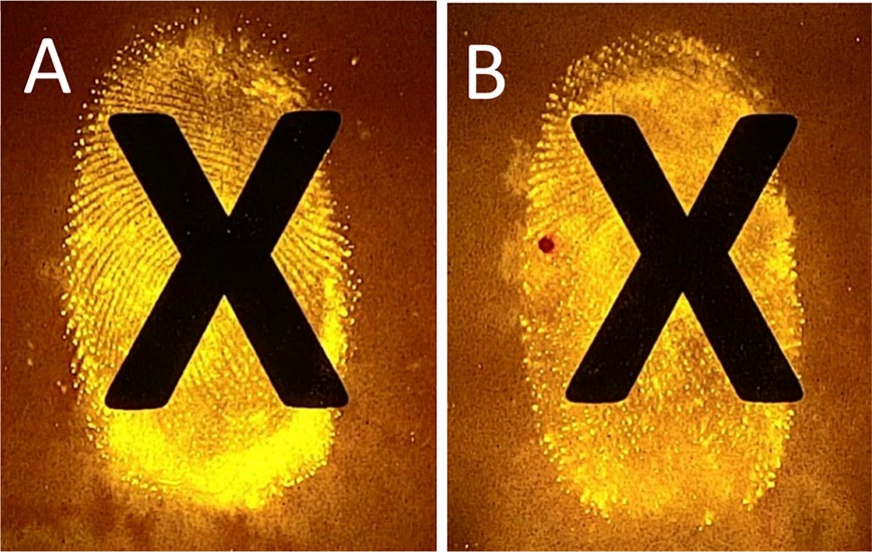
Fingermarks developed using DFO placed prior to printing an X (**A**) and on top of a printed X (**B**).

Whilst this method clearly offers context and crucial new information to document evidence, it was important to ascertain if standard fingermark development methods could still be used following on from this technique. Whilst this technique does, on occasion, provide excellent fingermark ridge detail, the consistency between samples is lacking when compared to traditional porous fingermark development methods. However the non-invasive nature of the gelatin lifting technique ensures this method could be used in conjunction with these techniques. The traditional development technique can still provide specific identification of a fingermark, whilst the technique described here can provide evidence of chronology without detriment. Figure 4 shows the same fingermarks seen in Fig. 3, but on the original paper substrate, developed using DFO. When the gelatin lifts were taken prior to development of the paper using ninhydrin, DFO or 1,2-Indandione, no detriment to the ridge detail developed on paper was observed. This was confirmed via split prints, fingermarks were placed on paper and a gelatin lift was taken of half of each of the fingermarks. Each fingermark was then developed on paper using ninhydrin, DFO or 1,2-Indandione, and no difference in the two halves of each fingermark could be seen for any of these reagents for any of the donors.

**Figure 5:**
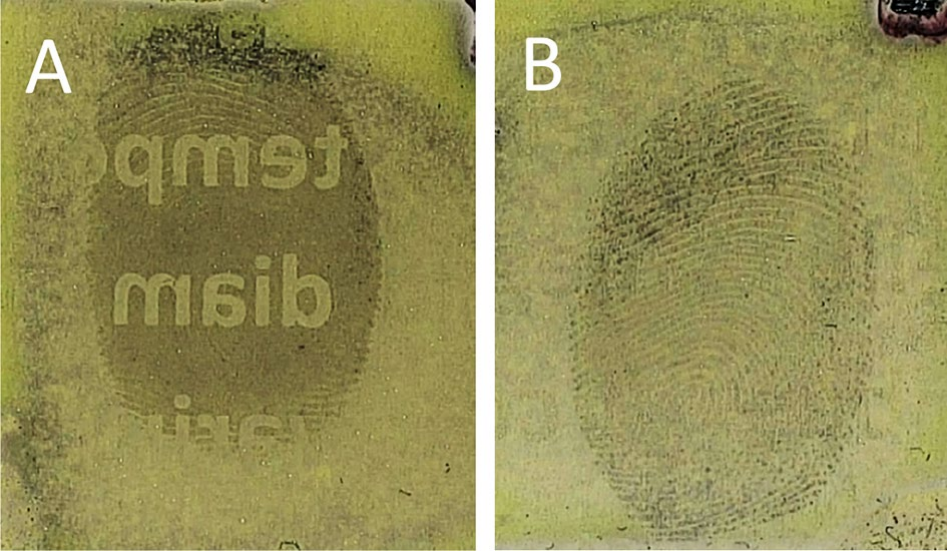
Gelatin lifted samples of fingermarks placed prior to printing prose (**A**), and on top of printed prose (**B**), developed in the RECOVER system.

As an even more realistic example, Fig. 5 shows the developed gelatin lifted samples of prose printed on top of a deposited fingermark (A) and a fingermark placed over printed prose (B).

## Discussion

The initial results in this investigation noted that the S_2_N_2_ vapours could develop fingermark residue on gelatin lifters, and said residue was visualised via the characteristic blue/black polymer formed^7^. This was true when a fingermark was placed directly onto the gelatin lifter surface, and when the lifter was used to lift from both porous and non-porous substrates. Because of this, further work was completed that intended to utilize these surface interactions in a manner that could provide important context regarding chronological deposition order of printed ink and fingermark deposits.

**Figure 6:**
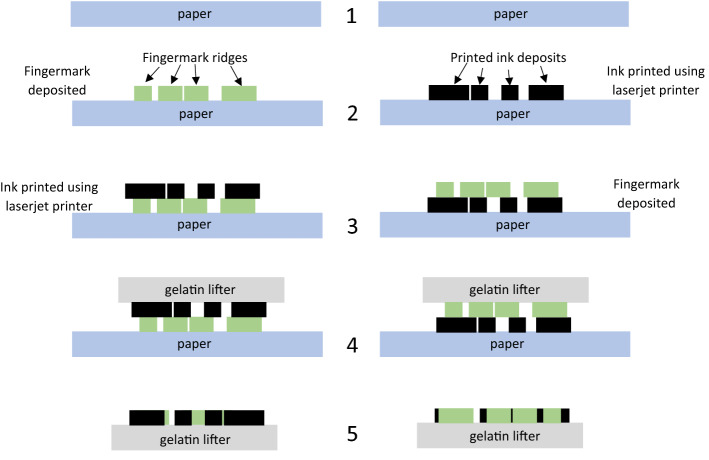
A cross sectional schematic showing the sequence of fingermark placement and printing prior to gelatin lifting.

As shown in Figs. 2, 3 and 5, the ink printed on top of the fingermark is clearly differentiated from the ink printed below the fingermark. In each case where the printed ink is on top of the fingermark deposit, no residues of said fingermark are transferred leaving a ‘gap’ that will react with the S_2_N_2_ vapour at the same rate as the gelatin lifter itself. This shows clear contrast with the areas where fingermark residues have been transferred from paper to gel surface. It is theorized that the reason for this occurrence, is because the fingermark residues have not been transferred from paper to gelatin lifter when ink is deposited on top of the fingermark itself. The ink is obstructing the fingermark components and thus preventing the characteristic polymerisation of S_2_N_2_ to (SN)_x_ that would enable visualisation of the fingermark itself. This is the same process as seen with the block of ink but on a more detailed scale, and in a more realistic example of real life document evidence types. The difference observed where the fingermark was placed over the printed text is arguably even more stark, as only the fingermark itself is visible. The smaller text size is further reducing interactions between ink and fingermark residue. As a result, the polymerisation in this case is still possible and the fingermark can be visualised. The schematic in Fig. 6 highlights this process in action and point 5 within this figure notes how toner detail may be visible through fingermark residue due to the gaps caused by fingermark ridges.

As previously discussed, there are several fingermark development techniques available to forensic practitioners for use on porous substrates^1^. In the majority of cases these techniques would be preferential for merely identifying a fingermark deposit. The work conducted in this study aimed to work in tandem with these traditional fingermark development techniques, allowing for chronology determination without detriment to the fingermark deposit development. Figure 4 shows that fingermarks may still be developed via traditional methods after gelatin lifting and, in doing so, yields no detriment to the quality of the fingermark deposit itself.

Whilst in the majority of cases traditional techniques would be preferential, there are instances where these may not be suitable due to their invasive nature. Due to the porosity of the surface and the fingermark permeating the substrate upon which it is deposited, the fingermark reagent must similarly permeate the surface and react with the fingermark components, enabling visualisation. The presence of ridge detail in some of the RECOVER developed gelatin lifted samples suggest that this technique may provide an alternative means of development that could be used when traditional techniques may be deemed unsuitable, such as those which may be of historical importance^3^. More work will be needed to assess the suitability of this method.

Much research involving complex analytical methods such as SIMS has been conducted in order to provide additional information to fingermarks found, such as chronological sequencing of paper handling and printing^15^. There are major limitations to any previous methods to determine paper handling sequencing, such as only working when specific fingermark reagents are used^11^. None of the previously proposed methods are amenable recommended to forensic examiners due to the numerous limitations they possess. The work outlined in this proof-of-concept study will require further investigation to fully elucidate the boundaries of its operational capacity, whereby larger sets and pseudo-operational trials may be undertaken. It is also worth noting, that whilst the RECOVER was used in this study with a porous material (gelatin lifter), the vacuum level required for the system to run as intended, meant that the quantity of gelatin lifter utilised per experiment was limited. Alternative lifting processes could be investigated further in this case. Despite these limitations however the facile method outlined in this paper serves to provide clear evidence for a potential technique which could easily be used to obtain information that could be vital to a case.

## Conclusion

The results of this research provide clear evidence that this essential information can be determined by utilising already established methodology and technology in an innovative way. Combining this with the previously acknowledged benefits of using gelatin lifters to maintain a non-invasive methodology, offers a highly beneficial and novel forensic method.

The primary focus of the work discussed here has been completed using a laserjet printer, since laser printed documents are far more common in everyday use. Further work may extend to the analysis of inkjet printers and the effectiveness of this new technique with inkjet printer deposition mechanisms. The ability to sequence different deposition methods (stamps, written ink etc.) can also be investigated, as well as greater analysis of the level of detail lifted. Studies into developing a method that would allow for processing larger surface areas would also be greatly beneficial and are ongoing.

Determining handling sequencing has long been seen as the ‘holy grail’ for forensic examinations. The research outlined within this paper offers forensic practitioners a simple technique that provides clear evidence regarding the timing of deposits on document evidence.
